# Pastourelle Narratives of Sexual Violence and Consent in Middle French and Middle Dutch Printed Popular Song

**DOI:** 10.1007/s11061-026-09881-3

**Published:** 2026-08-28

**Authors:** Cécile de Morrée

**Affiliations:** Faculty of Arts, Radboud University, Nijmegen, The Netherlands

**Keywords:** Middle French literature, Middle Dutch literature, Pastourelle, Popular song, Print culture

## Abstract

This article examines late-medieval reconfigurations of the pastourelle in Middle French and Middle Dutch popular printed song. While the French courtly pastourelle, preserved in manuscripts intended for aristocratic audiences, has often been read as both a critique and a reinforcement of courtly codes of conduct, the genre acquired new meanings in the widely circulating printed books that reached the more socially diverse publics of late medieval cities. To enable the study of these divergent messages across divers cultural contexts, this article proposes conceptualizing the pastourelle not as a literary genre but as a recurrent narrative. This approach allows for a broader understanding of the tradition, in which the pastourelle narrative functioned as a familiar framework for exploring themes of power and gender in various ways, while also absorbing influences from contemporary song traditions. As a result, this article demonstrates how printed popular songs diverge from the courtly paradigm of class conflict, reshaping the pastourelle narrative to articulate both restrictive and empowering messages concerning sexual violence, justice and consent, particularly addressed to female audiences.

## Introduction

The pastourelle was perhaps one of the most prevalent genres of medieval lyric. It had its high in twelfth- to fourteenth-century France, composed and performed by troubadours and trouvères, but relevant texts are transmitted in many European languages, including Occitan, French, Spanish, Italian and German (Paden, 1987). The pastourelle typically relates the accidental encounter of a knight and a shepherdess, whom he seeks to seduce, in a pleasant ambiance of natural greenery. This narrative, relating a cross-class encounter with various outcome, is considered distinctive of the genre, and it is most clearly present in Northern-French pastourelles (Spetia, 2012, p. 591; Bec, 1977-1978 I, pp. 119–136; Willaert, 2021, pp. 218–220). Most pastourelles are transmitted in manuscripts from courtly milieus, and were presumably composed by and for members of similar aristocratic environments. In accordance with their cultural background, pastourelles have been interpreted as at the same time critiquing and confirming the courtly codes of conduct, because the knight’s assertive and occasionally transgressive courtship practices are only temporarily permitted, by his stepping outside his courtly circle and into the shepherdess’s rural world (Gravdal, 1991; Leach, 2023; Smith, 2009; Zink, 1972).

In this article, however, I will move away from courtly culture and turn toward a very different but understudied sort of lyric, namely that of popular song, that flourished in late-medieval urban culture, and that was transmitted via affordable printed songbooks without music and, possibly, orally. These songbooks intended to appeal to a wide and diverse audience, including men and women of all social ranks, to ensure sufficient clientele to make the books commercially viable (Brown, 1963; De Morrée, 2023, 2024a, 2024b; Roper, 2015(b); Walsby, 2020; Pettegree, 2007; Salzberg, 2014; Grenby, Marazzi & Salman, 2021). Transported to such different surroundings, the pastourelle was disconnected from its double meaning of both critiquing and confirming courtly codes of conduct, to instead be blended in with the culture of the socially more varied population of the cities. I therefore contend that, in this context, the pastourelle narrative re-emerged as a familiar framework for new ideological messages about sexuality, foregrounding alternative norms and values.

Maintaining an orientation on the region that saw the courtly pastourelle’s heyday—Flanders, Artois, Hainaut, Brabant (Willaert, 2021, p. 218)—I will examine two songbooks that outshone competing collections both in popularity and volume. Printed in Paris (1535) and Antwerp (1544), they each include well over 200 songtexts, combining contemporary songs with fifteenth-century pieces, and their status as the most enjoyed collections of their time is confirmed by successive reprints (De Morrée, 2023, 2024a, 2024b; Van der Poel et al., 2004). Composed in Middle French and Middle Dutch respectively, they represent the two principal vernaculars of the region we now refer to as Northern France and the Southern Low Countries, which throughout the later Middle Ages functioned as a multilingual contact zone and fostered a thriving multilingual literary culture (Besamusca et al., 2025; Hugen, 2022; Kleinhenz & Busby, 2011; Morato & Schoenaers, 2019; Van de Haar, 2019).^1^ By the early sixteenth century, a well-developed international book-trading network was in place, connecting major printing centers like Paris, Antwerp, Frankfurt, and Lyon, and ensuring a lively commercial exchange of books between Paris and Antwerp in particular (Bellingradt et al., 2017; Pettegree, 2007; Walsby, 2016). In short, both songbooks likely circulated in both cities and beyond.

Despite their wide circulation, transitions from manuscript-based courtly genres to printed popular literature remain drastically understudied, especially when it comes to the role of French traditions in shaping Middle Dutch printed texts. Therefore, Bart Besamusca, Elisabeth de Bruijn, and Frank Willaert (2020, pp. 1–6, 93–124) emphasized the need for more cross-linguistic research on early printed narrative texts, given their distinctly transnational character. Pursuing this line of research is especially promising for the pastourelle, because, in contrast to the markedly European dimensions of the genre, there is no evidence of a Middle Dutch courtly pastourelle tradition of any significance (Willaert, 2021, pp. 228–233, 494). This absence is commonly attributed to the francophone orientation of the Flemish and Dutch courts, whose nobility favored French literature (Besamusca et al., 2020, pp. 7–8), prompting the question of how and why the pastourelle resurged in Dutch-language popular print culture.

This article’s objectives, therefore, are twofold. First, it seeks to close the chronological gap separating the extensively studied continental pastourelles of the twelfth through fourteenth centuries from their later medieval reiterations. Second, it interrogates the pastourelle’s influence upon literary traditions situated beyond the sphere of courtly or aristocratic manuscript culture, thereby reframing the discussion within the context of printed popular song. To address these considerations, I will answer two questions. The first question asks how we might formulate a definition of the pastourelle that enables its identification within late-medieval printed popular song. The second and principal question concerns why the diverse urban readership of subsequent centuries maintained an interest in the pastourelle. I argue that, although the popular songs examined here depart from earlier genre definitions of the pastourelle, they nonetheless retain its core narrative. However, departing from the courtly paradigm of class conflict, popular songs reshape that narrative to convey both restrictive and empowering messages about sexual violence and consent, that were better suited to a new audience in a changing world.

## The Pastourelle Narrative

In this article, I conceptualize the pastourelle as a narrative, because, in essence, it conveys a story, unfolding a sequence of events set in a particular place and conveyed through a narrator (Ryan, 2007). By choosing this elementary definition, I seek to do justice to the condensed nature of narrative songtexts, which rarely permit complex plot structures. This conciseness may itself be regarded as one of the pastourelle’s lyrical qualities, alongside its rhyme, stanzaic form, formulaic expressions, refrains, and performative dimension. In a similar vein, Christopher Callahan (2002, p. 8) describes the Old French pastourelle as ‘a forerunner of lyrico-narrative romance’, treating refrains as lyric insertions in a narrative structure.

Building on Ken Plummer’s (2019) understanding of narrativity as a cultural power, I specifically use the term ‘narrative’ to refer to a *recurrent* story pattern — a sequence of story-level events that reappears across diverse cultural forms. These forms may include literature, such as songs and romances; visual media, such as paintings or tapestries; dramatic performances and plays; or even non-fictional forms, like news reports or legal documents. Through their repeated circulation, narratives become embedded in culture, influencing how we structure thought and action and how we attribute meaning to experience. The medieval pastourelle was indeed such a recurrent story pattern, as is clear from the way its narrative circulated not only across courtly literary genres (Smith, 2009; Zink, 1972) and aristocratic tapestries (Baechle et al., 2022), but also through the appearance of its motifs and phrases in seemingly objective legal documents (Gravdal, 1991).

The pastourelle narrative’s essential core consists of three components, that remain stable across genres and over time. First, the narrative centers on an accidental encounter between two characters, one of whom expresses a sexual interest in the other. Second, the interaction is marked by a gendered social disparity, with the male character consistently occupying a superior rank. Third, the scene unfolds in a secluded natural environment — fields, forests, or rural paths. These three defining components persist in late-medieval printed songbooks, marking a distinct separation between pieces that perpetuate the courtly pastourelle and those that do not. Hence, they provide a solid foundation for further exploration.

Drawing on these fixed narrative components, the courtly story commonly depicts a chance encounter between a knight and a country maiden or shepherdess, succeeded by a spirited dialogue. The knight typically seeks to win her favor, though her response may vary, and his advances often become increasingly persistent as the narrative unfolds. They often end up having sex – either mutually voluntary or violently forced. In either case, the closing scene commonly depicts the girl expressing approval and encouraging the knight’s swift return.

Whereas Michel Zink (1972) understood the pastourelle primarily as a civilizational conflict between courtly and rural spheres, scholarship was reshaped by Kathryn Gravdal’s (1985, 1991) interpretation of the genre as a manifestation of gendered, class-based power dynamics, with sexual violence occurring in roughly one-fifth of Northern-French examples. Building on the work of both scholars, Geri Smith (2009) framed the central tension as one of power, ultimately casting the pastourelle as a critique of the social and sexual values of the courtly culture that produced it—a view that became widely accepted. Scholars like William Paden (1989), Evelyn Vitz (1997), and Elisabeth Leach (2023) proposed more positive interpretations of the violent scenes, viewing the French pastourelle as an illicit yet playful sexual fantasy—an imaginative erotic escape from the rigid codes of courtly conduct. Moving beyond the French tradition while retaining a focus on texts preserved in aristocratic manuscripts, Carissa Harris (2016, 2018) further shaped the field by interpreting selected Middle English pastourelles as sexual pedagogies for young women, from moral warnings to obscenity as a mode of sexual communication.

Recently, Leach (2023) has cautioned that recent scholarship tends to misrepresent continental courtly pastourelle traditions as uniformly violent, echoing Paden’s (1989) earlier warning not to overlook the genre’s characteristic range of outcomes. Most courtly pastourelles do not convey stories of sexual violence, even if they do reflect tensions associated with gendered social hierarchy. Many texts omit sexual encounters altogether, while others depict them as consensual. Some feature a resourceful girl who outwits the knight and escapes his control, while still others depict a group of shepherds banding together to drive the knight away. All these variations derived their significance from the audience’s recognition of the underlying narrative. In turn, the narrative enabled authors and performers to explore themes of power and gender in ways that could entertain, critique, or subvert expectations, while maintaining coherence within the tradition.

In this way, conceptualizing the pastourelle as a narrative helps address a challenge that has long confronted scholars: reconciling the pastourelle’s variation with the need for a workable and precise definition. This tension has resulted in definitions that are either too narrow (Zink, 1972), or too broad (Bec, 1977-1978). Many scholars have attempted to resolve this issue by introducing subcategories of pastourelle types, a strategy that has added further complexity to the definition (Leach, 2023). A more recent strategy is to characterize the genre as a debate poem, as a means of addressing the diversity of narrative outcomes (Baechle et al., 2022; Llewellyn, 2010).

Crucially, however, these earlier definitions are hard to reconcile with the pastourelle’s recurrence across cultural forms and periods, as their reliance on formal genre criteria obscures its reappearance in non-courtly contexts and varied textual forms. Scholars such as Zink (1972) and Bec (1977-1978) considered the presence of the so-called ‘chevalier-poète’—a male, first-person character-narrator—an essential element of the pastourelle. Similarly, Llewellyn (2010) and Baechle et al. (2022) identified dialogue or debate as the genre’s defining formal feature. However, the current analysis will reveal that Dutch popular pastourelles omit the ‘chevalier-poète’, while French popular pastourelles drastically reduce the amorous debate. Nevertheless, they all unmistakably recount the same narrative.

Conceptualizing the pastourelle as a narrative rather than a literary genre allows us to detach it from the formal features that have traditionally defined it. Releasing the pastourelle narrative from its formal constraints opens up a broader understanding of the tradition, one that situates printed popular song within the same narrative continuum as courtly lyric. This is relevant, for, as I will demonstrate, formal elements—like narration and dialogic structure—decisively shape how the narrative is interpreted across different literary contexts.

This approach furthermore requires examining popular songs not only for their diachronic continuity with the pastourelle tradition but also for their synchronic engagement with contemporary song culture. This culture flourished with the rise of print, marked by the proliferation of affordable vernacular songbooks across the European continent. Singing was a widespread pastime and printed songtexts addressed a broad range of themes, from religious devotion and military exploits to bawdy satire and love, thereby accommodating wide audiences. As a result of their extensive circulation, many songtexts share overlapping motifs and expressions, even when their narrative content diverges (De Morrée, 2023; Van der Poel et al., 2004).

Here, Paul Zumthor’s concept of ‘registre’ is useful (1963, pp. 144–150). First conceived to capture the interwoven network of literary motifs, stylistic features, and formulaic expressions that underpin courtly literature in its various manifestations, the notion of registre extends beyond the limits of genre. In popular song, motifs and expressions belonging to the courtly pastourelle registre reappear, such as fixed epithets for the girl (‘jeune’, ‘fillette’, ‘seullette’) or references to the rural landscape (‘buisson’, ‘champs’). However, as I will show, popular pastourelles also borrow formulaic language from the registre of adjacent popular song traditions, most notably in their representations of female sexuality. The concept of registre offers a productive framework for examining such literary elements that cannot be coherently analyzed within the rigid demarcations of genre.

In my analysis, I situate the popular pastourelles within their respective collections, on the assumption that each collection reflects motifs and expressions familiar to its intended audience. Building on the work of scholars like Judith Bennett (2002), Carissa Harris (2016, 2018), Hermina Joldersma and Dieuwke van der Poel (2009–2010), I follow their insight that various late-medieval vernacular songs conveyed moral lessons to young adults—especially women—promoting virtue, chastity, and sexual restraint. Within a single songbook, songs that share parallel expressions can therefore be read as participating in both a common registre and a shared (though not necessarily deliberately constructed) pedagogical framework that shaped values and ideas concerning sexuality.

## Pastourelle Reconfigurations in Printed Popular Song

Unsurprisingly, pastourelle narratives appear far less often in Dutch popular songs than in their French counterparts. The Paris Songbook includes six examples (songs P-56, P-86, P-88, P-140, P-153, P-173), whereas the Antwerp Songbook contains three (A-22, A-61, A-67).^2^ In these songs, sexual initiative consistently lies with the male character. His desire is reciprocated only once (A-61).

The most enduring element of the popular pastourelle is its opening scene, which depicts a chance meeting outdoors. As an exception, song P-173 shifts the encounter to its final stanza. The locations are typically rural, painted with phrases like ‘En l’ombre d’ung buisson’ (P-173, P-88), ‘parmy ces champs’ (P-88), ‘over Rijn’ (A-61), or ‘aen der heyde’ (A-22). Although P-56 begins within the civilized confines of a walled garden, the scene shifts to open countryside as the shepherdess attempts to escape her assailant. Only A-67 leaves the spatial setting unspecified, presumably to allow the legend of the assault of Saint Margaret of Leuven to be reshaped to fit the familiar contours of the pastourelle narrative. Significantly, most songs place the encounter *on the road*, underscoring both its accidental nature and the isolated setting.

Another recurring theme is the status gap between the two main characters. Beyond the obvious gendered hierarchy of medieval society, the songs hint at differences in wealth, social status, age and sexual experience. The girl is always portrayed as a young maiden; P-173 specifies that she is fifteen, while in A-61 her mother insists she is far too young to keep company with men. She is also, for instance, described as low-born (P-86, P-153) or as a shepherdess (P-56). By contrast, the male figure typically occupies a higher rank: he appears as a knight (A-61, A-22), a horseman (A-67), a student or learned man (P-140), or simply someone well-born (A-61, A-22) and wealthy (P-173, A-22). These contrasts foreground a dynamic of power and privilege, highlighting the persistence of the pastourelle narrative in popular song. A closer comparison of the Paris and Antwerp Songs, however, reveals a striking consistency in the distinct ways each group animates this core narrative.

### The Paris Songbook (1535)

Unlike the varied outcomes of the courtly pastourelle, all French popular pastourelles examined here involve some form of sexual coercion. The implementation of the pastourelle narrative as a rape narrative must therefore have been deliberate; the Paris Songbook’s compilers clearly intended to include stories of sexual violence. This is also evident from a comparison with an earlier edition of the songbook, which included a pastourelle where a clever shepherdess escapes a knightly aggressor (Jeffery, I, 1971, pp. 91–93). This song, however, was omitted in the 1535 edition.

The Paris songs are underpinned by a structural imbalance between male and female characters, that emerges through a combination of narratological strategies: a male character-narrator who offers only his account of events, a minimization of dialogue that deprives the female character of any real agency, and an overall pleasant tone that serves to legitimize the character-narrator’s perspective. By preserving this imbalance, the narrative reinforces male pursuit as an unquestioned norm: boys will be boys. Simultaneously, girls are consistently characterized as naïve, frivolous, driven by strong sexual desire, and unable to understand the potential consequences of sexual dally, including the loss of virginity, reputational harm or unintended pregnancy.

As in most courtly pastourelles, five out of six songs feature a chevalier-poète: a first-person male character-narrator, typically a well-born man, who mediates the story events but who also takes part in the story (P-56, P-86, P-88, P-140, P-153). Readers and listeners thus experience the scene entirely through his eyes, including his own involvement. He is usually introduced casually and cheerfully, traveling or wandering when he unexpectedly meets a solitary, attractive young woman. Song P-86, for instance, opens with ‘L’aultre jour my cheminoys, lalarigoy/ A Paris la bonne ville/ En mon chemin rencontray, la falarigoy/ Une jeune damoyselle honneste’, while P-153 narrates more concisely ‘En revenant de Sainct Anthoine en Brie/ Je rencontray une tresbelle fille’.

Furthermore, sexual initiative is consistently male and typically described using formulaic expressions that emphasize physical dominance. Common verbs include ‘prendre’ (to take or seize a woman) and ‘jeter’ (to throw her down). Sexual intercourse is framed in male-oriented terms, such as the fulfilment of his desire ([she:] ‘accomplir son desir’ (P-173)), his ‘doing’ to her a little bit of foolishness ([he:] ‘je luy fis la petite follie’ (P-153)) or by using verbs mimicking male movement, like ‘battre’ (poking (e.g. P-56)) and ‘lancer’ (stabbing or opening (P-86)). Consequently, the songs invite the audience to adopt the character-narrator’s perspective, fostering empathy with his motivations and desires. As a result, his persistent pursuit of the girl is framed in a notably sympathetic light.

This favorable image of the character-narrator is reinforced by the songs’ consistently pleasant atmosphere. In three songs (P-56, P-88, P-140) the encounter unfolds in idyllic settings, where nightingales sing and flowers bloom, evoked through formulaic expressions, such as ‘une fille, sur ma foy,/ A qui j’ay tout mon cueur donné,/ Au matinet à la rousée’ (P-56), and ‘En l’ombre d’ung buisonnet/ Chante le rossignollet/ Menant joyeuse vie’ (P-88). This idyllic setting aligns perfectly with the registre of French medieval love lyric. In these particular narratives, however, such descriptions become problematic. Drawing on the language of love songs, they lend a sense of sincerity to the male protagonist’s desires — an effect that stands in stark contrast to his acts of sexual coercion.

To similar effect, in three other songs (P-86, P-153, P-173) a lighthearted tone trivializes the events. This tone is often considered a remnant of the dancing feasts some pastourelles may have been part of (Spetia, 2012). It is especially evident in songs with musical refrains, which soften the bawdiness of the text and obscure the gravity of the knight’s actions. For example, P-86’s protagonist narrates with enthusiasm: ‘Et luy lansay en la playette, oette-oette, hin-hin, gué-gué, guin-guin!’ (I stabbed her in her small wound). Significantly, Christopher Callahan (2002) observed that in Old French pastourelles, narratives evolving around rape, abuse or seduction are often paired with similar refrains, encouraging the male protagonist, whereas songs about female resistance typically do not contain any refrains. In short, the male-centered, legitimizing use of the pastourelle rape narrative in popular song was influenced in several ways by earlier literary traditions.

Most unusually, however, the dialogue between the two main characters is drastically reduced. In some songs, the female character does not speak at all. This is noteworthy given the general prevalence of dialogic structures in early sixteenth-century popular songs. Vestiges of this practice persist in songs A-61 and P-153, both of which incorporate extended mother-daughter exchanges about sexual maturation and restraint. Moreover, limiting dialogue negates the pastourelle’s traditional function as a debate on seduction, sex, and consent with varied endings. With dialogue and its suspended outcome removed from the narrative, the girl’s faith ceases to be contested. Instead, the songs depict her yielding as certain, thereby legitimizing coercion.

Let us examine more closely the manner in which the events unfold. When the character-narrator first encounters the girl, he may ask her whether she is interested in a romantic relationship. In song P-153, his initial attempt is presented indirectly (‘Je luy demanday s’elle seroit m’amye’), while the second is expressed through direct speech, which conveys a heightened sense of urgency on his part ([he:] ‘“Serez vous m’amyes?”’). In two other songs, the same question appears in nearly identical wording, articulated either in direct speech (P-86) or indirect speech (P-56).

The girl in P-153 refuses, but this refusal is voiced only through the character-narrator (‘Elle my respond qu’elle estoit trop petite’), underscoring her lack of agency. Immediately afterward, the account shifts to physical coercion. She is thrown onto the grass, undressed, and subjected to abuse without being granted a single spoken line (‘Je la gettay sur l’herbette jolye/ Je luy levay son corset, en apres sa chemise/ Et je luy fis la petite follie’). This silencing is not incidental but structurally embedded, as evidenced by song P-86, which closely mirrors both the wording of her refusal and the subsequent assault. Furthermore, in P-86 the girl’s integrity in refusing is undermined by the portrayal of her motives as insincere: she intends to take the veil in the Huslieu convent, a place frequently cited in satirical literature (Jeffery, II, 1976, p. 225). As Kathleen Llewellyn (2010) noted in her analysis of the *débat amoureux* in the French courtly pastourelle and the sixteenth-century *Heptaméron*, seductive verbal duels construct a semblance of equality between the knight and the shepherdess, with both parties equally invested in the exchange. This sense of equality, however, is entirely erased in the popular pastourelles in the Paris Songbook.

In two songs (P-88, P-173), pre-sexual dialogue is entirely absent; the male character acts without negotiation, driven solely by desire. In P-88, the girl is denied any opportunity to refuse or consent, as the narrator immediately seizes her by the breast. Significantly, in four songs (P-56, P-88, P-140, P-173) the girl is referred to as ‘fillette’, a term meaning ‘girl’ but historically also associated with prostitution (Haemers, 2021).

Only in P-140 does the girl articulate her rejection across several verses. Responding to the male character-narrator’s greeting, she firmly dismisses him, requesting that her virginity remain intact (‘“Mon pucellaige my lairrez”’). He disregards her refusal and assures her he will not use force (‘“Nulle force ne vous feray”’), yet adds that he will embrace her ‘only a little’ (‘“Mais ung peu vous acolleray”’). This gesture suggests coercion, as the verb ‘accoller’ can denote either an affectionate hug or a forceful grip. The scene becomes more complex when he claims she embraced him in return – a statement that may reflect either a male erotic fantasy or a female involuntary reaction, as no consent is given. Her status is further diminished when he offers the ‘fillette’ the sum of ten francs, before he throws her onto the grass, a motive that is also common to the courtly pastourelle.

The girl in P-56 is subordinated through her portrayal as prey in a hunt, relentlessly pursued by the male protagonist. The character-narrator leans on hunting metaphors, boasting that she will be taken ‘à la pipée’—a bird-catching technique using glue. The girl eventually faints and is therefore unable to respond or consent. The character-narrator, however, exploits her silence and presses on regardless (‘je l’embrassé/ Tant que cuiday qu’elle cheust pasmée’).

When she wakes and realizes what has happened, she speaks for the first time in the song. Surprisingly, she expresses a longing for his embrace ([she:] ‘“Avant qu’il soit soleil couchant/ Entre voz bras seray gisant”’). Likewise, despite the preceding events, three other songs (P-88, P-153, P-173) conclude with the girl requesting an immediate renewal of intimacy, invariably articulated through the verb ‘recommencer’: (e.g. ‘“Recommancez s’il vous plait,/ Je suis toute ravie”’ (P-88)).

This recurrent scene raises the question of how a character subjected to evident coercion could so rapidly and willingly alter her stance. Possibly, the songs’ ambivalence contributed to their attraction, fostering awareness and stimulating discussion (Baechle et al., 2022; Colton, 2022; Saltzstein, 2017). This interpretation aligns with an urban setting, where popular songs were performed and enjoyed as communal activities on varied occasions (Brown, 1963; De Morrée, 2023; Freedman, 2006; Pleij, 2020; Strohm, 1985; Van der Poel et al., 2004, 2016). However, a close reading of the pastourelle songs in dialogue with the broader collection in the Paris Songbook reveals a particular understanding of girlhood that, to a contemporary audience, may have served as an explanation of this scene.

The most telling example is song P-173. What sets this song apart is its use of an omniscient narrator and its granting of a lengthy speech to the female character. Upon becoming pregnant, a young girl seeks to reassure her distressed mother. Rejecting any sense of calamity, she expresses confidence that the man responsible will remain loyal and provide financial support. However, the song offers several indications that the girl’s confidence is misguided and that the man has little intention of honoring his promises.

To start with, she describes what happened in formulaic, male-oriented terms: he grabbed her undershirt and took her virginity (‘“En pregnant ma chemise/ Il my depucella”’). Likewise, she describes the act of sex using the conventional vocabulary of male-centered pastourelles (‘“Sur son lict m’a gectée,/ Me fist à son plaisir.”’). Finally, she concludes with a familiar expression of discontent, as he was unwilling to engage in another round (‘“Je fus mal contente/ Qu’il ne recommença”’). Even though she claims to have slept with him voluntarily (‘“Je fus deliberée”’) and insists that he never hurt her (‘“Jamais ne me blessa”’), these formulaic expressions reveal the narrative’s underlying male perspective.

This male viewpoint can be attributed to the omniscient narrator, who reveals himself in the final stanza, where the song’s author-composer is introduced. The addition of such singer’s stanzas to popular songs was a common practice; however, these elements function as literary constructs rather than reliable sources of historical information (De Morrée, 2023). In this case, the song is framed as originating from a male persona, closely aligned with the male character-narrators of the other songs: he is a nobleman (‘Ung gentil compaignon’) who happened upon the girl in the woods. Consequently, the narrative voice is intimately connected to the male protagonist type found elsewhere, though it remains concealed behind a façade of female direct speech. The audience, familiar with such songs, would certainly have recognized the deceitful nature of the wellborn pastourelle seducer.

The audience would also have been familiar with the portrayal of young girls as naive, as this stereotype runs through the Paris Songbook. Girls and young women are recurrently depicted as imprudent, easily swayed, and negligent in safeguarding their chastity. In P-153, for instance, the girl ignores her mother’s belated counsel on sexual restraint, naively assuming that her unintended pregnancy will have few consequences because the man responsible has given her a powder said to aid postnatal recovery. Whatever its precise nature, the remedy’s narrative function is clear: it highlights her credulity and her vulnerability to an assailant who convinces her that the repercussions of an unwanted pregnancy can be effortlessly erased. Another example appears in P-86, where the girl admits she suffers from smallpox (‘la verolle’), understood here as a sexually transmitted disease (see also songs P-22, P-115). Because the illness signifies prior licentious behavior and an unchecked sexual appetite, these traits are implicitly projected onto her. The male character-narrator further ridicules both her and himself when he realizes he has been sowing onto old codpieces (‘vieilles brayettes’) rather than onto new linen (‘drap neuf’). More pertinent examples appear throughout the songbook, like song P-11, in which a reckless girl mindlessly trades her virginity for cheese, only to regret it when a cat eats it.

Most significantly, the girl in P-173 conforms to this stereotype by closing her speech with the promise ‘“Jamais je ne m’en tiendray”’. What she refuses to conform to remains unstated here, but is clarified by another song, P-176, which portrays a licentious young girl losing her virginity and honor because she cannot restrain herself. Using the same expression (‘Jamais elle ne s’en tiendra’ (P-176)), the song’s refrain repeats that once a girl has experienced sex, her desire becomes insatiable and she will never be able to maintain self-control. Moreover, the mothers in both songs are represented alike, as they respond identically to their daughters’ sexual awakenings: ‘Et sa mere brait et crie’.

In this way, song P-173 employs fixed formulae expressing recurring ideas that permeate the Paris Songbook. Particularly, the myth of uncontrollable female sexual appetite may have served to validate the girl’s retrospective consent: young women were considered incapable of regulating sexual arousal once stimulated. From this belief, it follows almost naturally that a girl would ask for more after being deprived of her virginity. Disturbingly, it also functions to legitimize abuse, reinforcing the harmful idea that all girls will learn to like it, and teaching men to disregard rejection.

### The Antwerp Songbook (1544)

The Antwerp songs convey a markedly different message. They recast the pastourelle narrative to privilege the female perspective, foregrounding her experience and asserting female agency.

All three Dutch popular pastourelles open with a focus on the female protagonist: she ventures out and unexpectedly meets an unknown man. Details in two songs suggest that the young maiden is seeking some form of diversion and stands on the threshold of sexual maturity (e.g. ‘vermeyden’, ‘roode rooskens wou si plucken,/ die aen der heyden staen’ (A-22), ‘met haer sneewitte handen’ (A-61)). The third song, A-67, recounts the fate of Saint Margaret of Leuven while adapting the narrative to fit the pastourelle framework. According to local legend, young Margaret (‘Margriete’) was raped and murdered by an angry band of thieves on September 2nd in the year 1225. Her body was cast into the Dijle river yet miraculously drifted upstream and returned to the city of Leuven (Joldersma & Van der Poel, 2009–2010; Putter, 2004). Across all three songs, the female figure is consistently foregrounded as the narrative’s principal character.

In keeping with this emphasis, the female characters speak extensively. Dialogue constitutes a central structural feature, with male and female voices alternating stanza by stanza to ensure a balanced exchange of viewpoints. Moreover, all three songs are narrated by an omniscient voice that does not participate in the events but maintains a fairly neutral position. The Antwerp songs, therefore, are free from the predetermined male perspective that permeates the Paris Songbook.

Another telling difference lies in how the atmosphere, after the first encounter, grows tense and perilous. Songs A-22 and A-67 (Margaret’s legend) are both stories of rape, employing strikingly similar language. The girl’s path is suddenly blocked by a stranger. At this point, it becomes clear that something ominous is about to unfold. The use of a question gives this moment a sense of vivid immediacy, drawing the audience’s attention and inviting them to inhabit her point of view: ‘Wat vant si in haren wege?/ Een welgheboren man’ (A-22) (What did she encounter blocking her way? A well-born man).

In both songs, the perpetrator openly declares his malicious intentions, while the girl firmly asserts her refusal. Much like his French counterparts, the man in A-22 acts immediately, taking off the girl’s clothes. The key difference, however, lies in her response. She immediately protests, insisting that he return her clothing at once (‘“Nu ghevet mi mijn cleyder,/ Och welgheboren man!”’). In reply, he explicitly threatens her with rape, which would cost her her virginity (‘[he:] “U eere suldy hier laten”’). This remark carries particular weight given her vulnerable position; she is standing before him naked. Similarly, the virtuous Margaret in A-67 is immediately ordered to yield to the thief blocking her path — a command she flatly rejects (‘[she:] “Uwen wille en doen ic niet!”’).

The moment of sexual union is described using familiar phrases borrowed from the registre of Dutch popular love songs – expressions the poets and their audiences knew well and that served as culturally recognized markers of intimacy. Typically, the man takes his lover by the hand, leading her to a quiet, inviting place, whether under a tree or behind closed doors. In the pastourelles, however, the maidens respond by raising their voices in protest. As soon as she’s back on her feet, the knight in A-22 sends her home. With unmistakable indignation, she declares that she has nowhere left to go. Returning home would mean facing her family in disgrace. Without hesitation, she lays the blame squarely on the knight, confronting him with fearless resolve: ‘[she:] ‘‘Waer sal ic henen keren,/ Waer sal ic henen gaen?/ Hadt ghi mi maecht gelaten,/ Ghi hadt veel badt gedaen.’’’. The modest Margaret (A-67) rises in fierce protest as well, vowing vengeance and warning her assailant that her three brothers will come for him. These heroines clearly understand that responsibility for rape rests with the perpetrator, not the victim, and they demand that the men be held accountable for their crimes.

The next move lies with the men, whose socially superior status grants them the power to decide how they will respond. Remarkably, the knight in A-22 accepts responsibility. He presents the girl with a golden ring as a pledge of loyalty. This is a promise of considerable weight, for in the Low Countries such vows were deemed legally binding even without witnesses (Joldersma & Van der Poel, 2009–2010). The tale closes with the birth of a charming, curly-haired baby, an ending that echoes the joyful conclusions of several love songs included in the same collection (e.g. A-129, A-43). Clearly, the outcome is presented as a happy resolution for both sides. This resolution reflects contemporary law, which allowed rape survivors to claim financial compensation for an injury understood primarily in economic terms (Joldersma & Van der Poel, 2009–2010; Broers & Jacobs, 2005; Delameillieure, 2025; Kelleher, 2013). While loss of virginity severely limited a woman’s prospects, marriage restored her honor. Thus, the song not only urges men to take responsibility but also reminds women of their legal rights and encourages them to claim what is theirs.

As we know, young Margaret’s fate was less fortunate. Unmoved by Margaret’s resistance, the offender murders her in cold blood. The story, therefore, conveys a warning, urging young women to stay within the safety of their homes, as was noted by Joldersma and Van der Poel (2009–2010). However, when read alongside A-22, Margaret’s song also conveys a warning directed at men. By linking the pastourelle narrative to the murder of a saint, the song portrays rape as a crime of utmost cruelty, reserved for the most barbaric of men. Through this grim example, it calls men to conscience, challenging them to reflect on the standards of their own behavior. In the Antwerp Songbook’s view, perpetrators face a choice: to make amends where possible or be condemned for eternity.

The final song under consideration (A-61) is perhaps the most compelling example of the pastourelle as a dialogue about love and seduction in their many guises, foregrounding a young girl’s uncertainties about how to respond to the attention of an attractive stranger. It is also unique because the girl chooses to elope with the knight of her own free will. While the knight’s intentions are unmistakable, conveyed through conventional formulas of desire that recur throughout Dutch popular love songs, the girl is deterred by the threat of her father’s wrath, because the knight’s noble birth makes him an unsuitable match (‘[she:] “Crijschman, ghi zijt te hooch geboren./ Ic ontsie so seere mijns vaders toren”’). In other words, she recognizes that elopement cannot culminate in marriage, a reality she carefully factors into her choice.

Like many girls in medieval literature, she seeks her mother’s counsel. The mother, however, firmly forbids her daughter from going out with men for at least another year, insisting she is too young. Instead, she advises her to remain indoors and devote herself to spinning green silk. (‘[the mother:] “Och dochter, ghi zijt noch wel te cleyne,/ Ghi slaept noch wel een jaer alleyn!/ Tsoheyme so sult ghi blijven/ Ende spinnen die groene side.”’) This counsel is not simply an injunction to chastity; it reflects an underlying concern with sexual immaturity. The song recalls another in the same collection, portraying a young woman who, with confidence and deliberation, leads her lover to bed as she secures her hair with a green silk ribbon (A-10). In A-61, the mother evidently considers her daughter too young for such behavior and responds by locking her in.

Eventually, the onset of sexual maturity prompts the girl to escape through the window in pursuit of the knight. The omniscient narrator concludes by noting that, in doing so, she risked her life – implying that wagering her virginity might ultimately cost her dearly – yet leaves the final judgment to the audience. Ultimately, the song portrays its heroine as a young woman negotiating the emotions and responsibilities of adulthood, yet also as someone who, after deliberate reflection, claims the right to make her own choices.

## Conclusion

In the early printed popular songs of the Low Countries and Northern France, the courtly pastourelle narrative was transformed to serve new pedagogical agendas concerning sexuality, albeit in markedly different ways. The Paris Songbook urges young women to exercise restraint as the primary safeguard against rape, framing this advice through a male-oriented, misogynistic lens. Victims are blamed for their supposed naiveté, or the assault is justified by invoking myths of female lust. Female characters lack agency, and dialogue is minimized. Although pastourelles with positive outcomes circulated widely, none appear in this extensive collection. Contrarily, the Antwerp Songbook foregrounds the female perspective, presenting a range of responses to questions of rape and consent. Female protagonists raise their voices, their doubts and experiences treated seriously. Aware of their legal rights, they confidently shift blame to the perpetrator and remind men of their responsibilities.

The late-medieval Southern Low Countries have frequently been portrayed as relatively female-friendly (Kittel & Suydam, 2004; Paijmans et al., 2020; Blockmans & Neijzen, 1999) Therefore, local cultural attitudes may well have informed the representation of gender and morality in Dutch songs, supporting women’s near-equality to men by promoting positive rolemodels in the domain of female sexuality and mutual consent (De Morrée, 2024a; Pleij, 2020). Moreover, the Antwerp songs’ foregrounding of women’s rights resonates with recent studies that highlight the wide range of local legal options available to women in marital and personal matters (Delameillieure, 2025, p. 512–513; Donahue, 2007).

However, such portrayals do not fully explain the contrasting messages found in these particular songs. Misogynist misconceptions about the supposedly natural female inclination to licentiousness were widespread, as is evidenced by many songs in both songbooks (Joldersma & Van der Poel, 2009–2010). Likewise, the compensation through money or marriage of rape victims was a well-known solution to minimize damage in Northern France as well as the Southern Low Countries (Donahue, 2007). Moreover, we should remind ourselves that these songs may well have been enjoyed by the same, multi-lingual audience.

Instead, let us consider the various literary traditions that influenced these regions and raise the question of language: why was Dutch selected as the medium for songs about resilience and women’s rights? The answer may lie in the absence of a Dutch courtly pastourelle tradition of notable significance. A dependance on prior courtly traditions is well visible in the French printed pastourelles. In contrast, in the absence of closely related literary predecessors, Dutch song tradition embodied no registre particularly connected to the pastourelle narrative. Therefore, the use of the Dutch language permitted poets a blank canvas to explore the pastourelle’s possibilities in light of fifteenth- and sixteenth-century interests, including the growing position of women as readers (Kittel & Suydam, 2004; Paijmans et al., 2020; Pleij, 2020). It has often been observed how early printers, particularly in Antwerp, sought to attract buyers by simultaneously playing at both new tastes and traditional genres (Besamusca et al., 2020, pp. 49, 82, 93–124). The Dutch popular pastourelle perhaps can be positioned on this crossroads of the old, the new, and the female.

## Footnotes

1 While first published in the early sixteenth century, both songbooks are generally considered late medieval rather than early modern, based on their content and linguistic features.

2 Song numbering follows the modern editions (Van der Poel et al., 2004; Jeffery, II, 1976); readers are referred to these editions for consultation of the texts.

## References

[CR1] Baechle, S., Harris, C. M., & Strakhov, E. (2022). *Rape culture and female resistance in late medieval literature*. Pennsylvania State University Press.

[CR2] Bec, P. (1977-1978). La lyrique française au Moyen Age (XIIe- XIIIe siècles). Contribution à une typologie des genres poétiques médiévaux. Tome I: Études. Tome II: Textes . Picard .

[CR3] Bellingradt, D., Nelles, P., & Salman, J. (2017). *Books in motion in early modern Europe: Beyond production, circulation and consumption*. Palgrave Macmillan.

[CR4] Bennett, J. (2002). Ventriloquisms: When maidens speak in English songs, c. 1300-1550. In A. L. Klinck & A. M. Rasmussen (Eds.), *Medieval woman’s song cross-cultural approaches* (pp. 187–204). University of Pennsylvania Press.

[CR5] Besamusca, B., De Bruijn, E., & Willaert, F. (Eds.). (2020). *Early Printed Narrative Literature in Western Europe*. De Gruyter.

[CR6] Besamusca, B., Demets, L., & Hugen, J. (Eds.). (2025). *The multilingual dynamics of medieval literature in Western Europe, c. 1200–c. 1600*. Brepols.

[CR7] Blockmans, W., & Neijzen, T. (1999). Functions of fiction. Fighting spouses around 1500. In W. Blockmans & A. Janse (Eds.), *Showing status: Representation of social positions in the late Middle Ages* (pp. 265–276). Brepols.

[CR8] Broers, E. J. M. F. C., & Jacobs, B. C. M. (2005). “Hoe men procederen sal inden saken aegaende den dagen van vrouwe crachten”. Enkele aantekeningen over het middeleeuwse strafrecht van ’s Hertogenbosch. *Recht in geschiedenis. Een bundel bijdragen over rechtsgeschiedenis van de Middeleeuwen tot de hedendaagse tijd* (pp. 91–102). Davidsfonds.

[CR9] Brown, H. M. (1963). *Music in the French secular theater, 1400–1550*. Harvard University Press.

[CR10] Callahan, C. (2002). Hybrid discourse and performance in the Old French pastourelle. *French Forum,**27*(1), 1–22.

[CR11] Colton, L. (2022). “Going all the way with Marot”: Empowerment in the pastourelle motet “L’autrier m’esbatoie/ Demenant grant joie/ Manere.” In A. K. Grau & L. Colton (Eds.), *Female-voice song and women’s musical agency in the Middle Ages* (pp. 271–296). Brill.

[CR12] De Morrée, C. (2023). Fashions of old and new songs: French popular printed songbooks around 1535. *Book History,**26*(1), 1–47.

[CR13] De Morrée, C. (2024a). Seks, dwang en instemming in het Antwerps Liedboek: Een Middelnederlands scenario ter voorkoming van seksueel geweld. *Queeste,**31*(1–2), 52–83.

[CR14] De Morrée, C. (2024b). Innovating the sixteenth-century French popular songbook in Lyon: Denis de Harsy, women and Orion in 1538. *Quaerendo,**54*, 362–380.

[CR15] Delameillieuere, C. (2024). *Abduction, marriage, and consent in the late medieval Low Countries*. Amsterdam University Press.

[CR16] Delameillieure, C. (2025). Mapping medieval marriage: Canon law, regionalism, and the evolution of legal history. *Journal of Medieval History,**51*(4), 499–514.

[CR17] Donahue, C. (2007). *Law, marriage and society in the later Middle Ages: Arguments about marriage in five courts*. Cambridge University Press.

[CR18] Freedman, R. (2006). Clément Janequin, Pierre Attaingnant, and the changing image of French music, ca. 1540. In M. Rothstein (Ed.), *Charting change in France around 1540* (pp. 63–96). Susquehanna University Press.

[CR19] Gravdal, K. (1985). Camouflaging rape: The rhetoric of sexual violence in the medieval pastourelle. *Romanic Review,**76*(4), 361–373.

[CR20] Gravdal, K. (1991). *Ravishing maidens: Writing rape in medieval French literature and law*. University of Pennsylvania Press.

[CR21] Grenby, M., Marazzi, E., & Salman, J. (eds.) (2021). *Special Issue: European Dimensions of Popular Print Culture*, *Quaerendo* 51(1–2) .

[CR22] Haemers, J. (2021). Women and stews: The social and material history of prostitution in the late medieval Southern Low Countries. *History Workshop Journal,**92*, 9–50.

[CR23] Harris, C. (2016). Rape narratives, courtly critique, and the pedagogy of sexual negotiation in the Middle English pastourelle. *Journal of Medieval and Early Modern Studies,**46*(2), 263–287.

[CR24] Harris, C. (2018). *Obscene pedagogies: Transgressive talk and sexual education in late medieval Britain*. Cornell University Press.

[CR25] Hugen, J. (2022). Vernacular multilingualism: The use of French in medieval Dutch literature. *Neophilologus,**106*(2), 181–198.

[CR26] Jeffery , B. (1971-1976 ). *Chanson verse of the early Renaissance*. 2 vols. Tecla editions.

[CR27] Joldersma, H., & Van der Poel, D. (2009–2010). Across the threshold to maturity: Gender and mobility in the Antwerp Songbook. *Iterinaria* 8–9: 165–223

[CR28] Kelleher, M. (2013). Later medieval law in community context. In J. Bennett & R. Karras (Eds.), *The Oxford Handbook of Women and Gender in Medieval Europe. *Oxford University Press.

[CR29] Kittell, E. E., & Suydam, M. A. (Eds.). (2004). *The texture of society: Medieval women in the Southern Low Countries*. Palgrave Macmillan.

[CR30] Kleinhenz, C., & Busby, K. (eds.) (2011). *Medieval Multilingualism: The Francophone World and its Neighbours.* Turnhout: Brepols

[CR31] Leach, E. E. (2023). *Medieval sex lives: The sounds of courtly intimacy on the Francophone borders*. Cornell University Press.

[CR32] Llewellyn, K. M. (2010). At play in the fields and playing the field: The *débat amoureux* in the *pastourelle* and the *Heptaméron*. *Parergon,**27*(1), 104–124.

[CR33] Morato, N., & Schoenaers, D. (Eds.). (2019). *Medieval francophone literary culture outside France*. Brepols.

[CR34] Paden, W. D. (1987). *The medieval pastourelle*. 2 vols. Garland.

[CR35] Paden, W. D. (1989). Rape in the Pastourelle. *Romanic Review,**80*(3), 331–249.

[CR36] Paijmans, M., Dietz, F., Geerdink, N., Leemans, I., De Morrée, C., & Veldhuizen, M. (2020). Pathways to agency: Women writers and radical thought in the Low Countries, 1500-1800. *Intellectual History Review,**31*(1), 51–71.

[CR37] Pettegree, A. (2007). *The French book and the European book world*. Brill.

[CR38] Pleij, H. (2020). *Oefeningen in genot: Liefde en lust in de late Middeleeuwen*. Prometheus.

[CR39] Plummer, K. (2019). *Narrative power: The struggle for human value*. Polity Press.

[CR40] Putter, A. (2004). Fier margrietken: A medieval ballad and its history. In P. E. Bennett (Ed.), *The singer and the scribe. European ballad traditions and European ballad culture* (pp. 69–88). Brill.

[CR41] Roper, A. (2015). Poor man’s music? The production of song pamphlets and broadsheets in sixteenth-century Augsburg. In R. Kirwan & S. Mullins (Eds.), *Specialist markets in the early modern book world* (pp. 175–198). Brill.

[CR42] Ryan, M. (2007). Toward a definition of narrative. In D. Herman (Ed.), *The Cambridge companion to narrative* (pp. 22–35). Cambridge University Press.

[CR43] Saltzstein, J. (2017). Rape and repentance in two medieval motets. *Journal of the American Musicological Society,**70*(3), 583–616.

[CR44] Salzberg, R. (2014). *Ephemeral city: Cheap print and urban culture in Renaissance Venice*. Manchester University Press.

[CR45] Smith, G. L. (2009). *The medieval French pastourelle tradition: Poetic motivations and generic transformations*. University Press of Florida.

[CR46] Spetia, L. (2012). The pastourelle as a popular genre. In K. Reichl (Ed.), *Medieval oral literature* (pp. 581–600). De Gruyter.

[CR47] Strohm, R. (1985). *Music in late medieval Bruges*. Clarendon Press.

[CR48] Van de Haar, A. (2019). *The golden mean of languages. Forging Dutch and French in the early modern Low Countries (1540–1620)*. Brill.

[CR49] Van der Poel, D., Geirnaert, D., Joldersma, H., & Grijp, L. P. (Eds.). (2004). *Het Antwerps liedboek*. 2 vols. Lannoo.

[CR50] Van der Poel, D., Grijp, L. P., & Van Anrooij, W. (Eds.). (2016). *Identity, intertextuality, and performance in early modern song culture*. Brill.

[CR51] Vitz, E. B. (1997). Rereading Rape in Medieval Literature: Literary, Historical, and Theoretical Reflections. *Romanic Review,**88*, 1–26.

[CR52] Walsby, M. (2016). Plantin and the French book market. In M. McLean & S. K. Barker (Eds.), *International exchange in the early modern book world* (pp. 80–101). Brill.

[CR53] Walsby, M. (2020). Les étapes du développement du marché du livre imprimé en France du XVe au début du XVIIe siècle. *Revue D’histoire Moderne & Contemporaine,**67*(3), 5–29.

[CR54] Willaert, F. (2021). *Het Nederlandse liefdeslied in de middeleeuwen*. Prometheus.

[CR55] Zink, M. (1972). *La pastourelle: Poésie et folklore au Moyen Âge*. Bordas.

[CR56] Zumthor, P. (1963). *Langue et Techniques Poétiques à l’Époque Romane*. Librairie C. Klincksieck.

